# Supplementary material for: A Critical View of the Application of the APEX Software (Aqueous Photochemistry of Environmentally-Occurring Xenobiotics) to Predict Photoreaction Kinetics in Surface Freshwaters
Source: Molecules. 2019 Dec 18;25(1):9. doi: 10.3390/molecules25010009 (PMC7017383; doi:10.3390/molecules25010009)
Supplement: Supplementary file 1 [file molecules-25-00009-s001.zip › 3.2.4_gcc-4.4.0.zip!/3.2.4_gcc-4.4.0/3.2.4_gcc-4.4.0/doc/octave/html/interpreter/Polynomial-Interpolation.html]

Polynomial Interpolation - Untitled


Next: Miscellaneous Functions,
Previous: Derivatives and Integrals,
Up: Polynomial Manipulations


---

### 27.5 Polynomial Interpolation

Octave comes with good support for various kinds of interpolation,
most of which are described in Interpolation. One simple alternative
to the functions described in the aforementioned chapter, is to fit
a single polynomial to some given data points. To avoid a highly
fluctuating polynomial, one most often wants to fit a low-order polynomial
to data. This usually means that it is necessary to fit the polynomial
in a least-squares sense, which is what the `polyfit` function does.

— Function File: [p, s, mu] = **polyfit** (x, y, n)  
> Return the coefficients of a polynomial p(x) of degree
> n that minimizes the least-squares-error of the fit.
>
> The polynomial coefficients are returned in a row vector.
>
> The second output is a structure containing the following fields:
>
> R: Triangular factor R from the QR decomposition. X: The Vandermonde matrix used to compute the polynomial coefficients. df: The degrees of freedom. normr: The norm of the residuals. yf: The values of the polynomial for each value of x.
>
> The second output may be used by `polyval` to calculate the
> statistical error limits of the predicted values.
>
> When the third output, mu, is present the
> coefficients, p, are associated with a polynomial in
> xhat = (x-mu(1))/mu(2).
> Where mu(1) = mean (x), and mu(2) = std (x).
> This linear transformation of x improves the numerical
> stability of the fit.
>
>
>
> **See also:** polyval, residue.

In situations where a single polynomial isn't good enough, a solution
is to use several polynomials pieced together. The function `mkpp`
creates a piece-wise polynomial, `ppval` evaluates the function
created by `mkpp`, and `unmkpp` returns detailed information
about the function.

The following example shows how to combine two linear functions and a
quadratic into one function. Each of these functions is expressed
on adjoined intervals.

```
     x = [-2, -1, 1, 2];
     p = [ 0,  1, 0;
           1, -2, 1;
           0, -1, 1 ];
     pp = mkpp(x, p);
     xi = linspace(-2, 2, 50);
     yi = ppval(pp, xi);
     plot(xi, yi);
```

— Function File: yi = **ppval** (pp, xi)  
> Evaluate piece-wise polynomial pp at the points xi.
> If pp`.d` is a scalar greater than 1, or an array,
> then the returned value yi will be an array that is
> `d1, d1, ..., dk, length (`xi`)]`.
>
>
>
> **See also:** mkpp, unmkpp, spline.

— Function File: pp = **mkpp** (x, p)  
— Function File: pp = **mkpp** (x, p, d)  
> Construct a piece-wise polynomial structure from sample points
> x and coefficients p. The i-th row of p,
> p `(`i`,:)`, contains the coefficients for the polynomial
> over the i-th interval, ordered from highest to
> lowest. There must be one row for each interval in x, so
> `rows (`p`) == length (`x`) - 1`.
>
> You can concatenate multiple polynomials of the same order over the
> same set of intervals using p `= [` p1`;` p2`;
> ...;` pd `]`. In this case, `rows (`p`) ==` d `* (length (`x`) - 1)`.
>
> d specifies the shape of the matrix p for all except the
> last dimension. If d is not specified it will be computed as
> `round (rows (`p`) / (length (`x`) - 1))` instead.
>
>
>
> **See also:** unmkpp, ppval, spline.

— Function File: [x, p, n, k, d] = **unmkpp** (pp)  
> Extract the components of a piece-wise polynomial structure pp.
> These are as follows:
>
> x: Sample points. p: Polynomial coefficients for points in sample interval. p `(`i`, :)` contains the coefficients for the polynomial over interval i ordered from highest to lowest. If d `> 1`, p `(`r`,` i`, :)` contains the coefficients for the r-th polynomial defined on interval i. However, this is stored as a 2-D array such that c `= reshape (`p `(:,` j`),` n`,` d`)` gives c `(`i`,` r`)` is the j-th coefficient of the r-th polynomial over the i-th interval. n: Number of polynomial pieces. k: Order of the polynomial plus 1. d: Number of polynomials defined for each interval.
>
>
>
>
> **See also:** mkpp, ppval, spline.
